# An exploratory study on the mechanism of Huangqi Guizhi Wuwu Decoction in the treatment of neuropathic pain

**DOI:** 10.1002/ibra.12033

**Published:** 2022-05-05

**Authors:** Bo‐Yan Luo, Hong‐Su Zhou, Qiu‐Xia Xiao, Yu‐Qi He

**Affiliations:** ^1^ School of Pharmacy Zunyi Medical University Zunyi Guizhou China; ^2^ Department of Anesthesiology Affiliated Hospital of Zunyi Medical University Zunyi Guizhou China

**Keywords:** Huangqi Guizhi Wuwu Decoction, molecular mechanism, network pharmacology, neuropathological pain

## Abstract

Huangqi Guizhi Wuwu Decoction (HGWD) has a definite effect on neuropathic pain (NP), whereas the specific mechanism has not been elucidated. The components and targets in HGWD were collected and identified through System Pharmacology Database (Traditional Chinese Medicine Database and Analysis Platform). Genecards and Online Mendelian Inheritance in Man databases were used to search for NP‐related genes. The Venn diagram was drawn to get the intersection target. Cytoscape 3.8.0 software was used to construct the compound‐disease‐target‐pathway networks. STRING database was applied to analyze protein–protein interaction of potential targets. Kyoto Encyclopedia of Genes and Genomes (KEGG) and Gene Ontology (GO) analyses were used to identify the function of genes related to NP. Finally, molecular docking was performed to visualize the binding mode and affinity between proteins and active ingredients. According to the intersection target of the Venn diagram, the network graph is constructed by Cytoscape and the results show the five compounds, β‐sitosterol, (+)‐catechin, quercetin, Stigmasterol, kaempferol, and 15 genes (CASP3, FOS, GSK3B, HSP90AA1, IKBKB, IL6, MAPK8, RELA, ICAM1, SELE, ELK1, HSPB1, PRKACA, PRKCA, RAF1) were highly correlated with NP. KEGG and GO of 15 genes results that TNF, IL‐17 and MAPK signaling pathway were Significantly related to the pathological mechanism of NP. Molecular docking showed that core genes in this network were IL‐6 (TNF and IL‐17 signaling pathways), ICAM1 (TNF signaling pathway), and CASP3 (three signal pathways). This study found that the five active compounds, three core genes, and three signaling pathways may be the key to the treatment of NP by HGWD.

## Graphical abstract

The intersection genes of Huangqi Guizhi Wuwu Decoction (HGWD) and neuropathic pain (NP) were analyzed by the Kyoto Encyclopedia of Genes and Genomes and Gene Ontology to obtain the relevant pathway network map, and then the key genes in the signaling pathway were molecularly docked to explain the potential effect of HGWD in the treatment of NP.

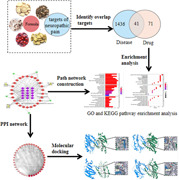

## 1 INTRODUCTION

Neuropathic pain (NP) is a chronic disease that sternly affects the patients' quality of life, which is marked by an incidence of 6.9%–10% in the general population.^1^ The cause of the disease may be that the peripheral nervous system or central nervous system releases a chemical medium that can increase sensitivity and stimulate pain receptors.^2^ The clinical manifestations are characterized as “burning pain,” “stinging pain,” “electrical shock pain,” and even “loss of local sensory or numbness,” which make the patients feel uncomfortable. The treatment tactics for NP include drug therapy^3^, ^4^ and nondrug therapy.^5^ Unfortunately, because of the complicated process of NP, no perfect therapeutic regimen is available so far, which makes the treatment of NP extremely challenging. Thus, it is imperative to explore the drugs and mechanisms for treating NP. For NP, traditional Chinese medicine (TCM) treatment may provide new ideas for its treatment.

TCMs are characterized by its significant therapeutic effect and relatively low toxicity and low cost, which has been achieved due to extraordinary advances in recent decades, such as arsenic trioxide used to treat acute promyelocytic leukemia.^6^ In clinical practice, the essence of TCM is compatibility between different TCM materials, which can show a synergistic therapeutic effect of enhancing curative effect or reducing toxicity.^7^ TCM is used to treat many diseases and has broad application prospects which may be an ideal therapeutic product alternative such as chronic diseases like diabetes and neurodegenerative diseases.^8^, ^9^ Huangqi Guizhi Wuwu Decoction (HGWD), one of the TCMs, was made by Zhang Zhongjing (Han Dynasty), which consists of five TCMs: Jujubae Fructus, Paeoniae Radix Alba, Hedysarum Multijugum Maxim, Cinnamomi Ramulus, and Zingiber Officinale Roscoe. From the perspective of TCM, HGWD is characterized by consolidating the surface without leaving the evil, dispelling the evil without hurting the right, and taking both the evil and the right into consideration. From the view of western medicine, HGWD has medicinal actions, such as anti‐inflammation, oxidation resistance, analgesia, and autoimmune reaction. It can be used for treating rheumatoid arthritis,^10^ protecting renal function,^11^ and treating frostbite of the shoulder joint.^12^ In addition, studies have reported that HGWD can significantly ameliorate the blood cycle, prevent neurotoxicity, and boost the rehabilitation of peripheral nerves.^13^ Moreover, it can also improve the expression of various neurotrophic factors, reduce the production of free radicals, and improve nerve function, which be used in the treatment of diabetic peripheral neuropathy.^8^ However, the most prominent characteristic of the TCM formula is its multiple compounds, multiple targets, and multiple signaling pathways, as well as its synthetic therapeutic effects, which exert distinctively preventive and therapeutic effects; thus, the application of TCM faces enormous obstacles.^14^

Consequently, if the action mechanism of TCM prescription for treating NP is explored through new tactics, it could be a great contribution to mankind.^15^ The network pharmacology method has advantages in the comprehensive analysis of multiple compounds and multiple targets. At present, the mechanism of action of various Chinese medicines has been discovered through network pharmacology methods.^16^, ^17^ Thus, the anti‐NP component and its potential targets in HGWD were decoded through network pharmacology during this study, which provides a new dimension to the study of the regime of HGWD in treating NP. In addition, we also explored their potential “component‐disease‐target‐pathway,” which will promote the research progress of candidate drugs for TCM.

## 2 MATERIALS AND METHODS

### 2.1 Identifying compounds in HGWD

First, the following two phytochemical databases: TCM Database and Analysis Platform (TCMSP) and TCMs Integrated Database (TCMID) were used to gather and discern the molecular components of five Chinese herbal medicines in HGWD.^18^

### 2.2 Screening of active compounds from HGWD

Oral bioavailability (OB) and drug similarity (DS) were performed to predict the possible effective compounds in HGWD. OB is the most momentous parameter in pharmacokinetics, which is used to reflect the speed, degree, and dosage ratio of drugs entering the blood circulation.^19^ DS is a comprehensive reflection of pharmacodynamic properties, which was used to evaluate whether a compound in a drug design is chemically suitable for the qualitative characteristics of the drug.^20^ The Absorption, Distribution, Metabolism, and Excretion (ADME) screening model of TCMSP was used to screen compounds with OB ≥ 30% and DS ≥ 0.18, which ultimately excluded unqualified compounds from the list.

### 2.3 Genetic targets of identified compounds in HGWD

The identified target genes of the active ingredients were collected from the UniProt^21^ database, which contains the gene name and gene ID corresponding to the active ingredient, and finally gains the herbal–ingredient–target interrelate data set of HGWD.

### 2.4 Disease target gene collection

The treatment target gene information of NP comes from the GeneCards database^22^ and Online Mendelian Inheritance in Man (OMIM) database.^23^ GeneCards database, which integrates numerous literature information, covers the analysis data of genes in multiple databases. It is a comprehensive database of human genes and includes any information related to genes. OMIM database has a collection of human disease information by classifying and naming diseases with genetic components and also has information regarding the relationship between phenotypes and related etiological genes.

### 2.5 Potential target prediction

A Venn diagram was created using the R software to show the overlap between the effective molecule and disease‐related genes. Thus, the bioactive compounds in the HGWD formula in NP treatment were obtained, and their potential targets were identified.

### 2.6 Pharmacological network construction

A pharmacological network is composed of nodes and edges. The entities constituting the network nodes are the networks of five TCMs of HGWD and their bioactive compounds, related targets, and related diseases. Cytoscape, an open‐source Java software,^24^ was applied to build a mesh of TCM and compounds, compounds, and targets.

### 2.7 Gene Ontology (GO) and Kyoto Encyclopedia of Genes and Genomes (KEGG)

In addition, the KEGG pathway^25^ was used to analyze HGWD‐ and NP‐related functional pathways. The OmicShare tool^26^ was applied to achieve GO analysis and visualize genes.

### 2.8 Protein–protein interaction (PPI) network

The STRING database^27^ was applied to identify PPI between selected target genes. Through PPI we can understand the interaction between protein and the working principle and the functional relationship of proteins in the biological system.

### 2.9 Molecular docking

To further elaborate on the interactions between molecules (potential compounds and potential targets) and to predict their binding mode and affinity, we selected five active compounds and three targets for molecular docking. From the Protein Data Bank database, we collected the three‐dimensional (3D) structure of the protein; the two‐dimensional (2D) structure of the ligand molecule was downloaded from the PubChem database; and ChemBio 3D software was used to calculate, minimize the molecular energy, and store as a 3D structure. The receptor protein was performed dehydration and removal ligand in Chimera 1.15 software, and executed hydrogenation and charge calculation with Autodock software. Then, setting the active pocket site of small molecules ligand binding, SeeSAR 11.2.0 was applied to, respectively, dock three proteins and five active compounds.

## 3 RESULTS

### 3.1 ADME screens the ingredients in HGWD

HGWD is composed of five kinds of TCMs (Jujubae Fructus, Paeoniae Radix Alba, Hedysarum Multijugum Maxim, Cinnamomi Ramulus, and Zingiber Officinale Roscoe), each Chinese medicine contains dozens or even hundreds of compounds. Through OB and DS, 790 compounds were screened by the ADME model (Table 1); after removing the duplicates, there were a total of 668 compounds, of which 63 compounds were listed in the candidate component library (Table 2).

**Table 1:** The list of the compounds of the five herbal species in HGWD.

Herbal name	Number of compounds
Jujubae Fructus	133
Paeoniae Radix Alba	85
Hedysarum Multijugum Maxim	87
Cinnamomi Ramulus	220
Zingiber Officinale Roscoe	265
Total	790

Abbreviation: HGWD, Huangqi Guizhi Wuwu Decoction.

**Table 2:** ADME screening of selected 63 compounds.

No.	Molecule name	MW	OB (%)	DL
1	Stepharine	297.38	31.54786691	0.33376
2	Spiradine A	311.46	113.5246051	0.60736
3	Zizyphus saponin I_qt	472.78	32.69113507	0.61923
4	Jujuboside A_qt	472.78	36.66570163	0.61915
5	Coumestrol	268.23	32.48702929	0.33733
6	Daechuine S6	548.75	46.48469652	0.79151
7	Daechuine S7	514.74	44.81774487	0.82806
8	Jujubasaponin V_qt	472.78	36.98963109	0.63448
9	Jujuboside C_qt	472.78	40.2624316	0.61911
10	Mauritine D	342.46	89.12509381	0.45286
11	Berberine	336.39	36.86124504	0.77665
12	(*S*)‐Coclaurine	285.37	42.35064217	0.23518
13	Ziziphin_qt	472.78	66.9452858	0.61926
14	Ruvoside_qt	390.57	36.12101953	0.75671
15	Malkangunin	432.56	57.71384384	0.62642
16	Stepholidine	327.41	33.10625074	0.54083
17	Nuciferin	295.41	34.43102883	0.40475
18	Protoporphyrin	562.72	30.86028979	0.55583
19	Fumarine	353.4	59.26250458	0.82694
20	21302‐79‐4	486.76	73.52245496	0.7661
21	Moupinamide	313.38	86.71215907	0.26454
22	β‐Carotene	536.96	37.18433337	0.58358
23	(−)‐Catechin	290.29	49.6763868	0.24162
24	Quercetin	302.25	46.43334812	0.27525
25	(3*S*,6*R*,8*S*,9*S*,10*R*,13*R*,14*S*,17*R*)‐17‐[(1*R*,4)‐4‐ethyl‐1,5‐dimethylhexyl]‐10,13‐dimethyl‐2,3,6,7,8,9,11,12,14,15,16,17‐dodecahydro‐1*H*‐cyclopenta[*a*]phenanthrene‐3,6‐diol	430.79	34.36766291	0.78147
26	11α,12α‐Epoxy‐3β‐23‐dihydroy‐30‐norolean‐20‐*en*‐28,12β‐olide	470.71	64.77389307	0.37586
27	Paeoniflorgenone	318.35	87.59312084	0.36678
28	(3*S*,5*R*,8*R*,9R,10*S*,14*S*)‐3,17‐dihydroxy‐4,4,8,10, 14‐pentamethyl‐2,3,5,6,7,9‐hexahydro‐1*H*‐cyclopenta[*a*]phenanthrene‐15,16‐dione	358.52	43.55620167	0.53276
29	Lactiflorin	462.49	49.12131675	0.79711
30	Paeoniflorin	480.51	53.87037516	0.78709
31	Paeoniflorin_qt	318.35	68.17576188	0.39507
32	Albiflorin_qt	318.35	66.64076901	0.32626
33	Benzoyl paeoniflorin	584.62	31.27447298	0.74612
34	Sitosterol	414.79	36.91390583	0.7512
35	Kaempferol	286.25	41.88224954	0.24066
36	Mairin	456.78	55.37707338	0.7761
37	Jaranol	314.31	50.82881677	0.29148
38	Hederagenin	414.79	36.91390583	0.75072
39	(3*S*,8*S*,9S,10*R*,13R,14*S*,17*R*)‐10,13‐dimethyl‐17‐[(2*R*,5*S*)‐5‐propan‐2‐yloctan‐2‐yl]‐2,3,4,7,8,9,11,12,14,15,16,17‐dodecahydro‐1*H*‐cyclopenta[*a*]phenanthren‐3‐ol	428.82	36.22847056	0.78288
40	Isorhamnetin	316.28	49.60437705	0.306
41	3,9‐di‐O‐methylnissolin	314.36	53.74152673	0.47573
42	5′‐Hydroxyiso‐muronulatol‐2′,5′‐di‐O‐glucoside	642.67	41.71766574	0.69251
43	7‐O‐methylisomucronulatol	316.38	74.68613752	0.29792
44	9,10‐Dimethoxypterocarpan‐3‐O‐β‐d‐glucoside	462.49	36.73668801	0.9243
45	(6*aR*,11*aR*)‐9,10‐dimethoxy‐6*a*, 11*a*‐dihydro‐6*H*‐benzofurano[3,2‐*c*]chromen‐3‐ol	300.33	64.25545452	0.42486
46	Bifendate	418.38	31.09782391	0.66553
47	Formononetin	268.28	69.67388061	0.21202
48	Isoflavanone	316.33	109.9866565	0.29572
49	Calycosin	284.28	47.75182783	0.24278
50	FA	441.45	68.96043622	0.7057
51	(3*R*)‐3‐(2‐hydroxy‐3,4‐dimethoxyphenyl) chroman‐7‐ol	302.35	67.66747949	0.26479
52	Isomucronulatol‐7,2′‐di‐O‐glucosiole	626.67	49.28105539	0.62065
53	1,7‐Dihydroxy‐3,9‐dimethoxy pterocarpene	314.31	39.04541112	0.47943
54	(−)‐Taxifolin	304.27	60.50621692	0.27342
55	(+)‐Catechin	290.29	54.82643405	0.24164
56	*ent*‐Epicatechin	290.29	48.95984114	0.24162
57	Taxifolin	304.27	57.84156034	0.27345
58	Peroxyergosterol	428.72	44.39151838	0.82
59	β‐Sitosterol	414.79	36.91390583	0.75123
60	6‐Methylgingediacetate2	394.56	48.73489111	0.32202
61	Stigmasterol	412.77	43.82985158	0.75665
62	Poriferast‐5‐*en*‐3β‐ol	414.79	36.91390583	0.75034
63	Dihydrocapsaicin	307.48	47.07062881	0.19251

Abbreviation: ADME, Absorption, Distribution, Metabolism, and Excretion; DL, drug similarity; OB, oral bioavailability; MW, molecular weight.

### 3.2 Active compounds and potential target genes related to NP

**Figure 1:**
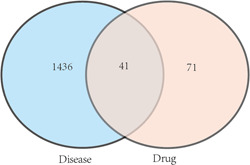
Venn diagrams of 63 active ingredient target genes of five Chinese herbal medicines and NP disease target genes. [Color figure can be viewed at wileyonlinelibrary.com]

**Figure 2:**
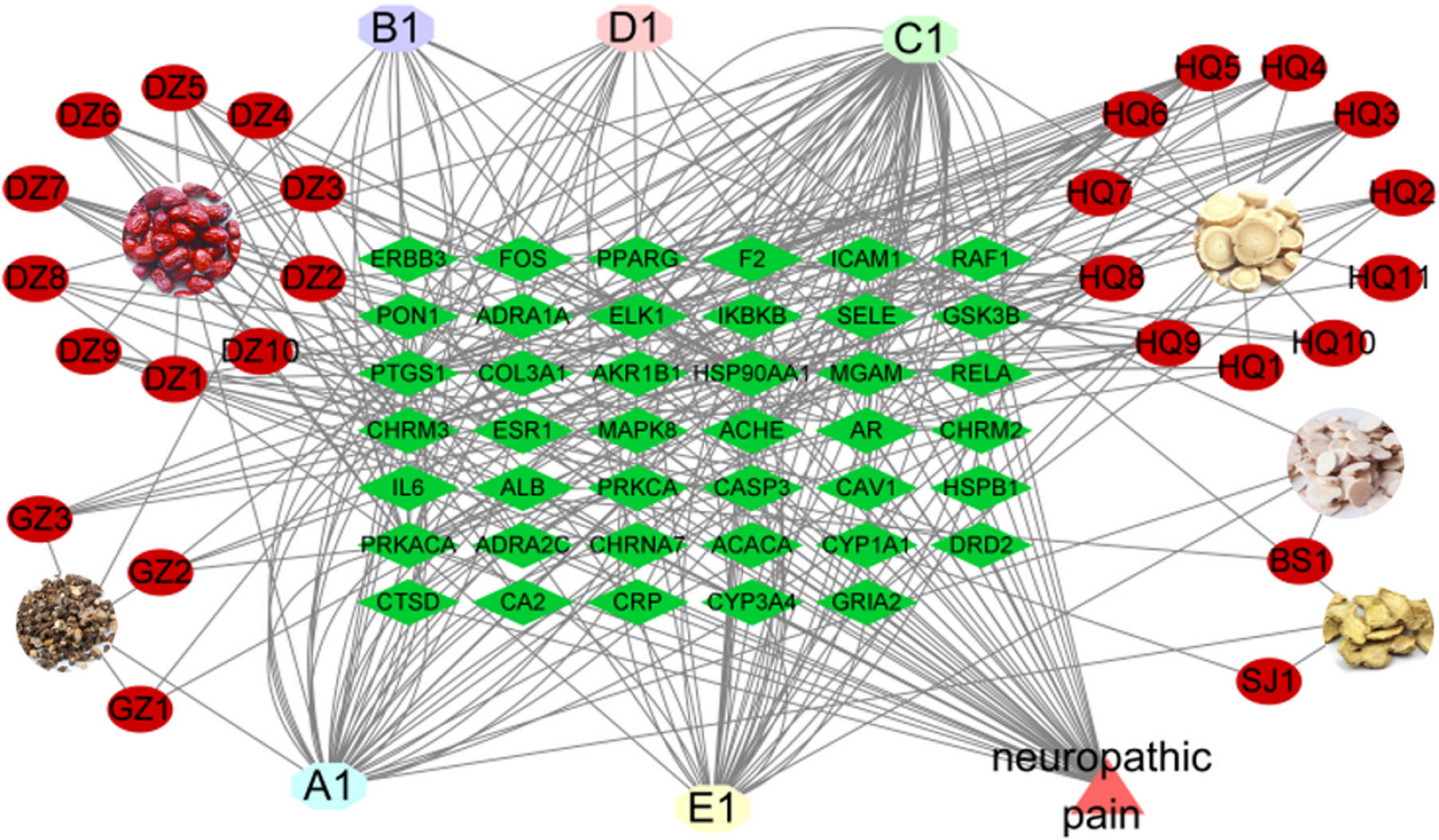
Molecule‐target gene–disease graph. Five kinds of Chinese herbal medicines were represented by orange ellipses, 41 target genes were represented by green rhombus, and 31 active compounds were represented by the remaining. A1, β‐sitosterol; B1, catechin; C1, quercetin; D1, stigmasterol; E1, kaempferol. [Color figure can be viewed at wileyonlinelibrary.com]

Using the UniProt database, 63 compounds contained 112 genes, of which 41 were potential target genes, that is, the intersection between 63 compounds and disease‐related genes (Figure 1). These 41 potential target genes correspond to 31 active compounds (Table 2), of which 5 are common components of HGWD, that is, β‐sitosterol(A1), (+)‐catechin(B1), quercetin(C1), Stigmasterol(D1), kaempferol(E1) (Table 3). Notably, β‐sitosterol was examined in four components of HGWD; (+)‐catechin was tested in three different herbs; and quercetin, stigmasterol, and kaempferol were examined in two different herbs (Table 3). What is interesting is that the five common components detected in the HGWD both contribute a lot to NP (Figure 2). A TCM‐molecule‐disease‐gene network graph was built using Cytoscape 3.8.0, including 78 nodes and 376 edges (Figure 2). The abbreviated information of the 31 compounds in Figure 2 (which contain the common components of the five traditional Chinese medicines) is in Table 4.

**Table 3:** Common active ingredients of five Chinese medicines.

Drug				Compounds
DZ	GZ	BS	SJ	β‐Sitosterol (A1)
DZ	GZ	BS		(+)‐Catechin (B1)
DZ	HQ			Quercetin (C1)
DZ	SJ			Stigmasterol (D1)
HQ	BS			Kaempferol (E1)

**Table 4:** Thirty‐one active compounds related to potential target genes.

No.	ID	Molecule name
1	A1	β‐Sitosterol
2	B1	(+)‐Catechin
3	C1	Quercetin
4	D1	Stigmasterol
5	E1	Kaempferol
6	DZ1	Stepharine
7	DZ2	Coumestrol
8	DZ3	Mauritine D
9	DZ4	Berberine
10	DZ5	(*S*)‐coclaurine
11	DZ6	Stepholidine
12	DZ7	Nuciferin
13	DZ8	Fumarine
14	DZ9	β‐Carotene
15	DZ10	(−)‐Catechin
16	BS1	Paeoniflorin
17	HQ1	Jaranol
18	HQ2	Hederagenin
19	HQ3	Isorhamnetin
20	HQ4	3,9‐di‐O‐methylnissolin
21	HQ5	7‐O‐methylisomucronulatol
22	HQ6	(6*aR*,11*aR*)‐9,10‐dimethoxy‐6*a*,11*a*‐dihydro‐6*H*‐benzofurano[3,2‐*c*]chromen‐3‐ol
23	HQ7	Bifendate
24	HQ8	Formononetin
25	HQ9	Calycosin
26	HQ10	FA
27	HQ11	1,7‐Dihydroxy‐3,9‐dimethoxy pterocarpene
28	GZ1	(−)‐Taxifolin
29	GZ2	*ent*‐Epicatechin
30	GZ3	Taxifolin
31	SJ1	6‐Methylgingediacetate2

### 3.3 Bioinformatics analysis

**Figure 3:**
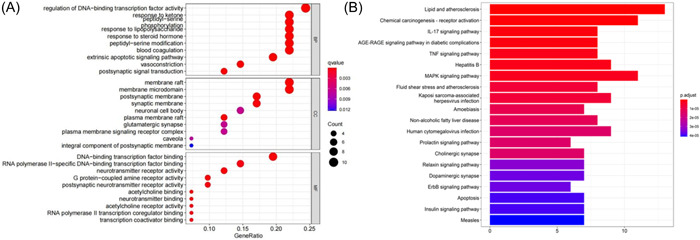
Gene Ontology (GO) enrichment analysis and Kyoto Encyclopedia of Genes and Genomes KEGG pathway of the intersecting genes. (A) The *y*‐axis represents the “biological process” categories that are significantly enriched relative to target genes in GO, and the *x*‐axis represents the enrichment scores of these targets. The quantitative results of GO enrichment analysis are only the top 10 participating Biological Process (BP), Cellular Components (CC), and Molecular Function (MF). (B) The *y*‐axis represents the pathway and the *x*‐axis represents the enrichment fraction of these targets. *p* Value represents significance; *p* < 0.05 represents the smaller the *p* value; the redder the color, the higher the significance. [Color figure can be viewed at wileyonlinelibrary.com]

**Figure 4:**
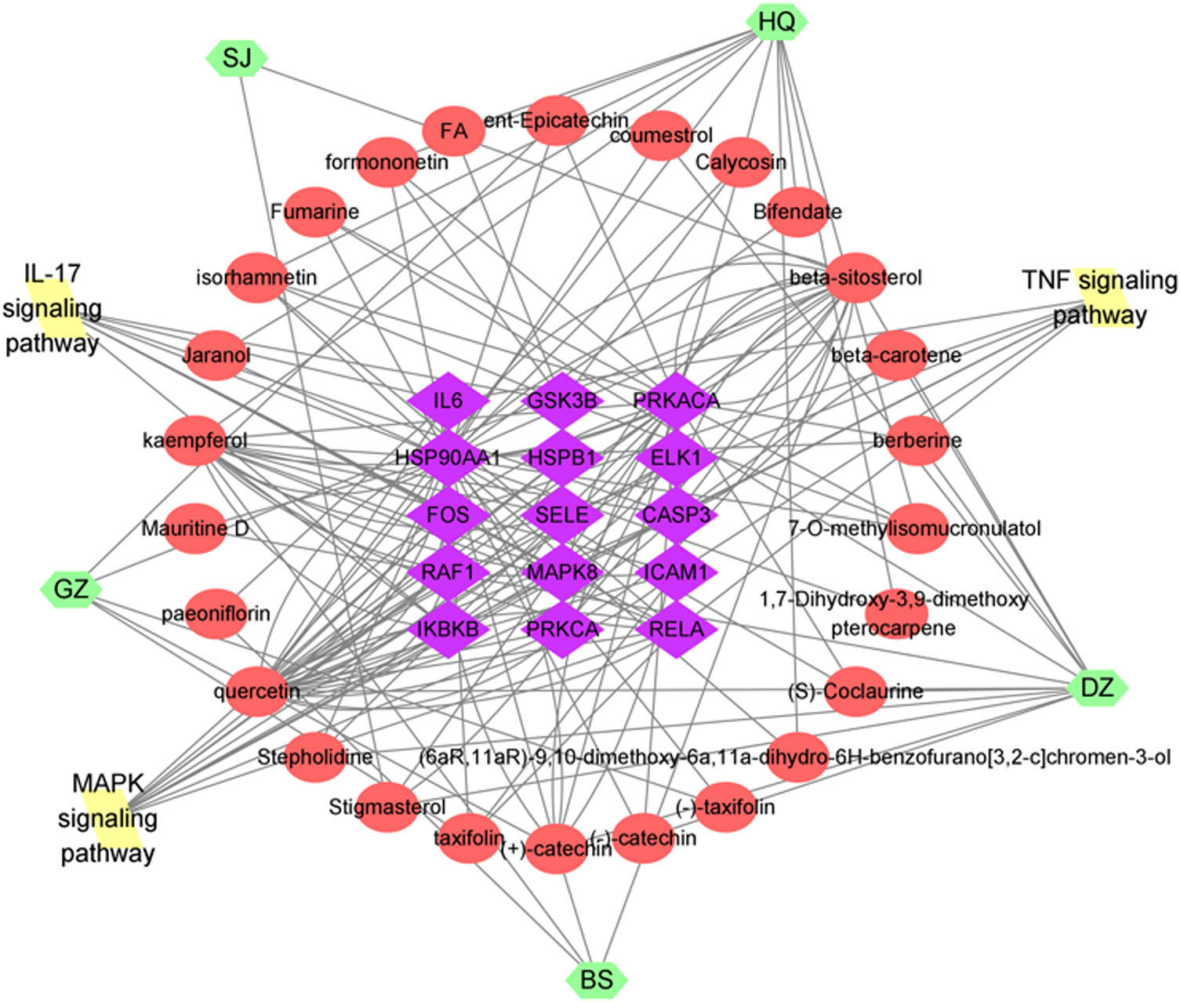
The target–pathway network for HGWD. Herbs are represented by green hexagons, red spheres indicate compounds, green diamonds indicate target genes, and paths are marked by yellow quadrilaterals. GZ, Cinnamomi Ramulus; HQ, Hedysarum Multijugum Maxim; SJ, Zingiber Officinale Roscoe; BS, Paeoniae Radix Alba; DZ, Jujubae Fructus. [Color figure can be viewed at wileyonlinelibrary.com]

To discern the action and related signal channel of pivotal genes, GO and KEGG pathway enrichment analyses were applied. In GO enrichment analysis, we listed the top 10 BP, MF, and CC processes of the target genes (Figure 3A). In addition, we listed the top 20 signaling pathways related to the above 41 potential target genes (Figure 3B). The results found that the core pathways containing TNF, MAP kinase (MAPK), and IL‐17 signaling pathways, which were underlying remedial processes in NP, 15 genes were related (Table 5), which may be the key target genes involved in HGWD treatment of NP (Figure 4). Chinese medicine‐compound‐gene‐pathway contains 49 nodes and 164 edges.

**Table 5:** The three signaling pathways are related to 15 target genes.

No.	Target name
1	CASP3
2	FOS
3	GSK3B
4	HSP90AA1
5	IKBKB
6	IL6
7	MAPK8
8	RELA
9	ICAM1
10	SELE
11	ELK1
12	HSPB1
13	PRKACA
14	PRKCA
15	RAF1

*Note*: The three signaling pathways are the MAPK, IL‐17, and TNF signaling pathways.

### 3.4 PPI network of target genes

**Figure 5:**
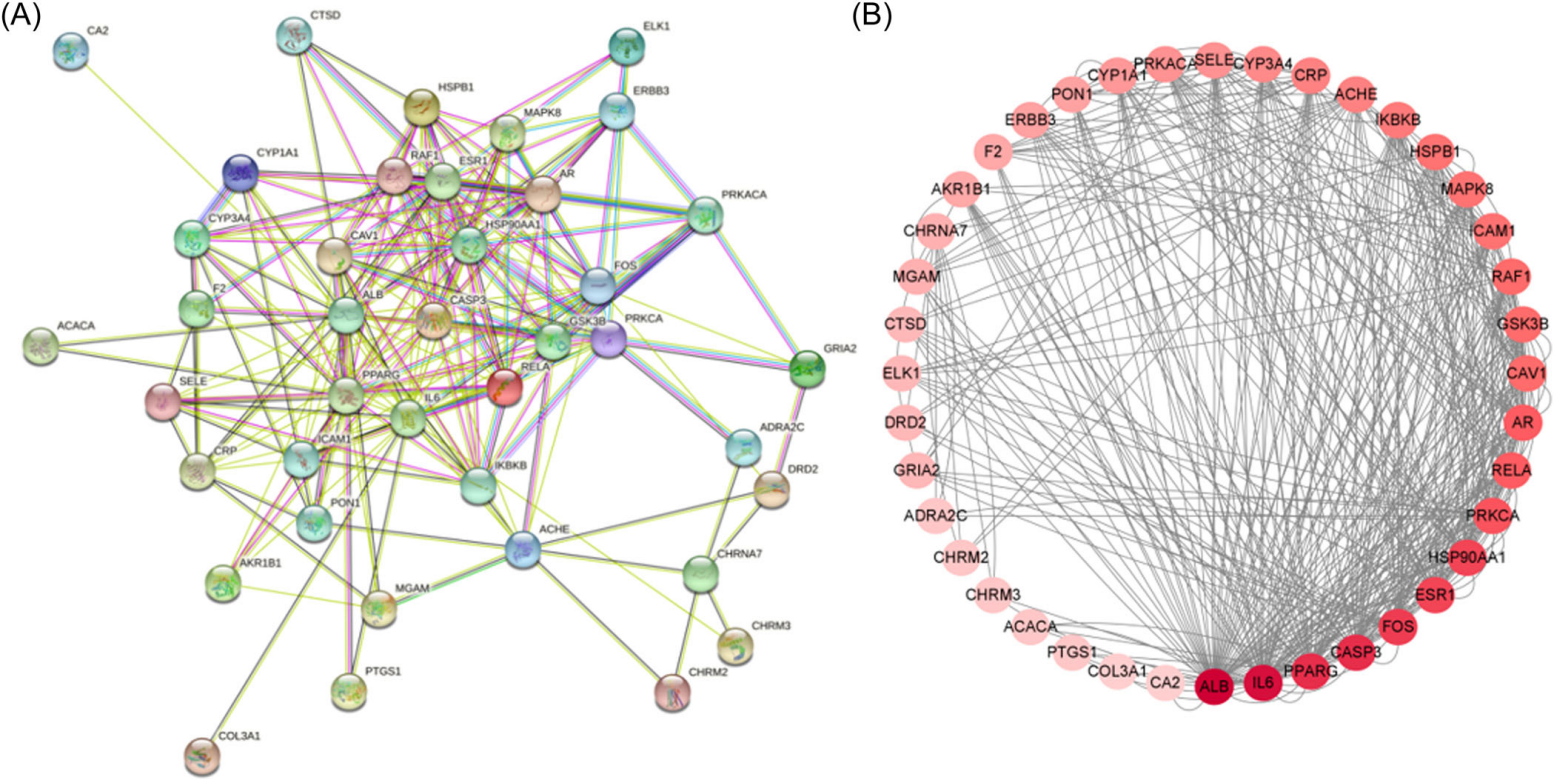
Protein–protein interaction (PPI) network diagram. (A) The colors of the lines have different meanings, where sky blue and plum red lines represent known interactions. Green, red, and blue represent predicted interactions. Grass green, black, and lavender represent others. (B) The PPI network contains 41 points and 420 lines. [Color figure can be viewed at wileyonlinelibrary.com]

The PPI relationship of 41 target genes was obtained by using the STRING tool, and the PPI relationship network diagram obtained by selecting a comprehensive score of >0.4 has 41 nodes and 210 edges (Figure 5A). To decipher the core genes of the 41 target genes, we used Cytoscape 3.8.0 to visualize the 41 target genes, and the resulting PPI network graph contains 41 points and 420 lines. Besides, the color of each point is a direct ratio to the level of its participation in the network (Figure 5B). The results show that the main core genes in the network include IL‐6, CASP3, and intercellular adhesion molecule‐1 (ICAM1), which are involved in inflammation‐related pathways, that is, TNF, IL‐17, and MAPK signaling pathways (Table 5 and Figures 4 and 5B).

### 3.5 Molecular docking of five active compounds and three core protein

**Figure 6:**
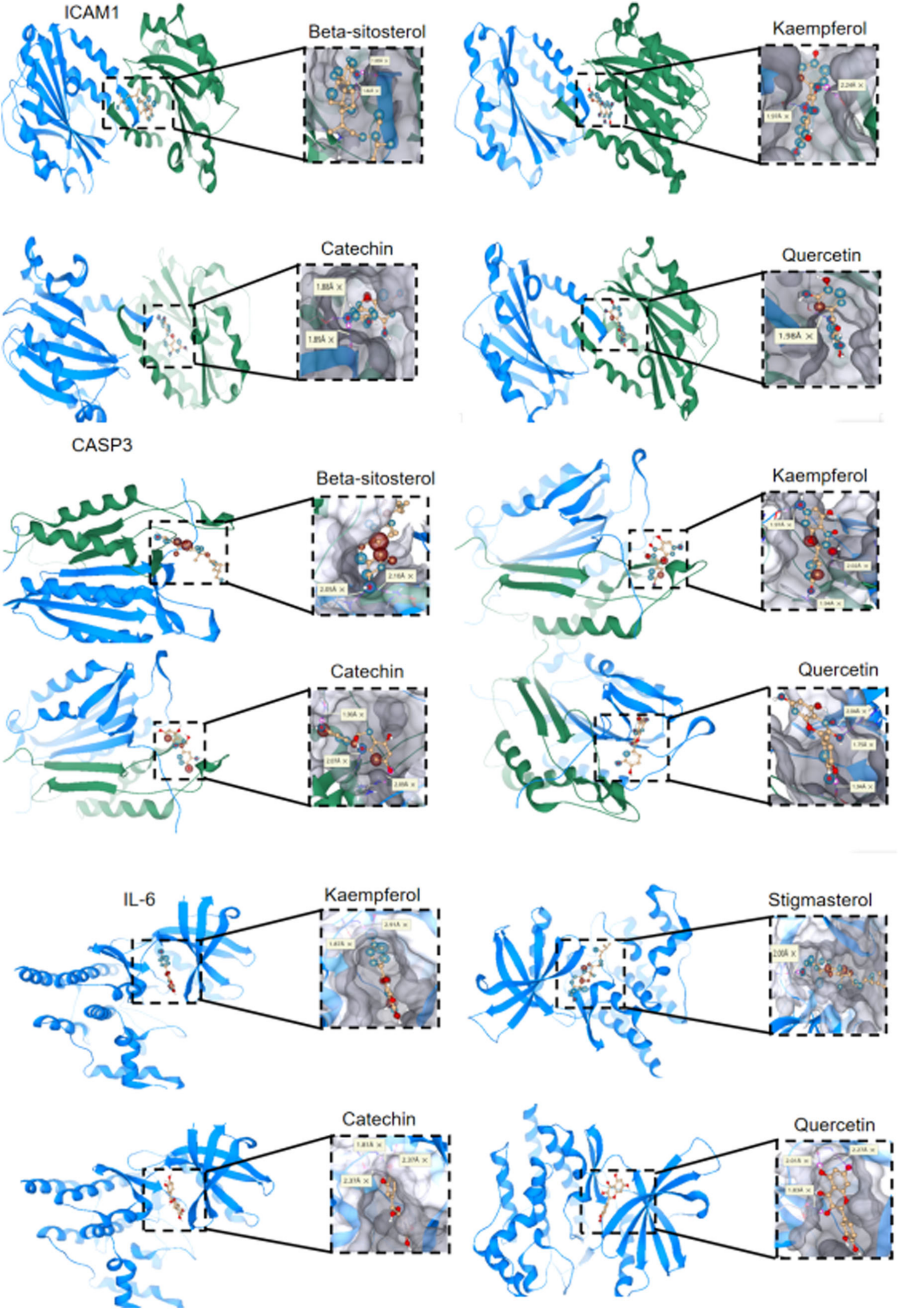
ICAM1, CASP3, and IL‐6 docking diagram. The colored balls represent hydrogen bonds, and the yellow markers represent the hydrogen bond distance between two atoms. [Color figure can be viewed at wileyonlinelibrary.com]

Molecular docking showed that the five active compounds of β‐sitosterol, catechin, quercetin, stigmasterol, and kaempferol can easily enter and bind to the active pockets of IL‐6, CASP3, and ICAM1 proteins (Figure 6). IL‐6 can be docked with four compounds, namely kaempferol, stigmasterol, catechin, and quercetin, with scores of −6.2, −5.9, −5.4, and −5.8, respectively. While CASP3 can dock with β‐sitosterol, catechin, kaempferol, and quercetin, with scores of −6.6, −6.2, −6.2, and −6.5, respectively. ICAM1 can also be docked with β‐sitosterol, catechin, kaempferol, and quercetin with the scores of −6.7, −6.0, −6.2, and −6.7, respectively.

## 4 DISCUSSION

Using network pharmacology and public database to unearth widespread text, pharmacokinetic factors screening, target prediction, and bioinformatic analysis were applied to decode the effective molecules, latent targets, and mechanisms of HGWD in treating NP. From the TCM‐molecule‐disease‐gene network figure, we concluded that the five common components have more network lines, that is, the contribution is large. Then, 15 key genes that NP intersected with 5 common components were obtained, and three pathways most related to the 15 key genes were explored by KEGG and GO, result that the Chinese medicine‐compound‐gene‐pathway map was made. Finally, molecular docking result that three of the 15 genes, IL‐6, ICAM1, and CASP3, can be molecularly docked with four of the five common components with preferable docking scores. So, we mainly discuss these five common components, three signaling pathways, and three genes, which may be the key to the treatment of NP.

### 4.1 The five common components

β‐Sitosterol, the common compound of the four components in HGWD except for Hedysarum Multijugum Maxim, has been detected by our application of network pharmacology, which has a wide range of anti‐inflammatory effects. It can reduce the expression of pro‐inflammatory factors and pro‐inflammatory enzymes by inhibiting the activation of the p38, ERK, and NF‐kB pathways induced by Lps, thereby inhibiting the activity of microglia to reduce inflammation.^28^ It also mediates the anti‐inflammatory effects of activated neutrophils in mice through l‐type voltage‐dependent calcium channels, intracellular calcium, and PI3K activity in a time‐ and dose‐dependent manner.^29^ β‐Sitosterol is considered a potential anti‐NP drug^30^ with beneficial anti‐inflammatory and analgesic effects. Among the 41 targets screened out of 31 compounds, 15 genes were involved in pathways related to NP. β‐Sitosterol targets 4 of 15 genes, namely HSP90AA1, CASP3, PRKACA, and PRCA. Quercetin has antioxidation, anti‐inflammatory, immune protection, neuroprotection, anticancer, and analgesic effects.^31^, ^32^, ^33^ However, studies have shown that although quercetin has been shown to have anti‐inflammatory and immune effects in vitro cell experiments and in vivo animal experiments, these results are not supported in human studies.^34^ Interestingly, our results indicated that the potential target of quercetin was confirmed by network pharmacological analysis. Of the 15 genes in the NP‐related pathway,^10^ 12 are potential targets of quercetin, which are IL6, CASP3, FOS, HSP90AA1, RELA, RAF1, HSPB1, PRKACA, ELK1, PRKCA, ICAM1, and SELE. Kaempferol is a common pharmacological component of Radix Astragali and Radix Paeoniae Alba. It can significantly inhibit MAPK‐related EPK and p38 pathways, which inhibits IL‐1 β‐stimulated inflammatory response.^35^ Kaempferol inhibits LPS‐induced TLR4/MyD88 inflammatory pathway, which relieves neuroinflammatory response and improves blood–brain damage.^36^ This study successfully identified eight anti‐NP targets belonging to kaempferol. Among them, IL6 and HSP90AA1 are the two most important targets.

Many foods and herbs contain catechins, and intake of catechins can help treat neurological and neurodegenerative diseases.^37^ Catechin inhibits different inflammatory mediators, which obstructs the activation of NF‐kB and MAPK.^38^ Stigmasterol has a neuroprotective effect comparable to resveratrol. It fights oxidative stress through the antioxidant defense mechanism of hydrogen peroxide reduction and attenuates oxidative stress by modulating SIRT1‐FoXO3a.^39^ In addition, related studies have reported that stigmasterol regulates GABA receptor subtypes to exert antianxiety and anticonvulsant effects and can be used as a new steroid compound for the treatment of neurological diseases.^40^

### 4.2 The three NP‐related signaling pathway

The MAPK signaling pathway plays a key role in the signal transmission of microglia under NP conditions. Inflammatory mediators, such as inflammatory factors and growth factors, produced through the MAPK signaling pathway will sensitize the nociceptive dorsal horn neurons through presynaptic and postsynaptic mechanisms, which result in persistent pain hypersensitivity.^35^, ^41^ Relevant studies have established the contribution of the MAPK signaling pathway in the development of neuroinflammation and neurotoxicity.^37^, ^42^ The MAPK signaling pathway can be used as a valuable target for the treatment of NP, and the established NP can be reversed with MAPK inhibitors.^41^

TNF is a pivotal member of the cytokine mediation system and can participate in systemic inflammation.^34^ It can be expressed in different levels of the nervous system when the nerve is injured, including nerve injury site, dorsal root ganglia, spinal dorsal horn, brain, and higher centers are all increased.^43^ Through TNF receptor, TNF‐α can regulate the release of glutamate, increase dendritic structure remodeling, synaptic connection strength, and enhance the role of glutamate excitatory synaptic transmission, which the excessive excitability of pain transmitted in neurons becomes mechanical hypersensitivity.^44^

Compared with the above two pathways, there are few reports on the role of the IL‐17 pathway in nerve pain regulation. Related research reports that IL‐17 promotes the persistence of tactile hypersensitivity after nerve injury by activating astrocytes and secreting pro‐inflammatory cytokines.^45^ In addition, new studies have shown that IL‐17/IL‐17R was involved in paclitaxel‐induced NP and the disorder of SOM^+^ neuron excitability and inhibitory synaptic transmission.^46^ These results suggest that the IL‐17 signaling pathway may provide new targets and novel ideas for the treatment of NP.

### 4.3 The three pathway‐related target genes

IL‐6 is a typical functional pleiotropic cytokine, which contributes to host defense when expressed normally.^47^ However, excessive IL‐6 is involved in the pathological process of arthritis,^10^ gastrointestinal inflammation,^48^ neuroinflammatory diseases,^37^ and so on. ICAM‐1 binds to the ligand lymphocyte functional antigen‐1, which induces inflammatory cell aggregation and promotes arthritis.^10^ ICAM‐1 is also closely related to neuroinflammation,^49^ CASP3 can participate in an important process of maintaining tissue homeostasis, and has been proven to be a key mediator of neuronal cell apoptosis. CASP3 poses a new challenge for determining the pharmacological approach for the treatment of many neurological diseases.^50^

HGWD contains important active compounds, such as quercetin,^32^, ^33^ kaempferol,^35^, ^36^, ^51^ and β‐sitosterol,^28^, ^29^, ^30^ which can effectively inhibit inflammation and oxidative stress. It protects nerves and relieves pain and other effects. HGWD has also been reported to potentially treat NP,^8^, ^13^ and our findings were similar. Although the content of our study is mainly focused on the presentation of the publication, it also further demonstrates the potential of HGWD in the treatment of NP. In addition, TNF, IL‐17, and MAPK signaling pathways^5^, ^43^, ^45^, ^46^ as well as IL‐6, ICAM1 and CASP3^47^, ^49^, ^50^ have also been reported to be related to NP treatment. The molecular docking of related neurodegenerative diseases also confirmed this.^52^ Thus, we speculate that quercetin, kaempferol, β‐sitosterol, and stigmasterol are active ingredients regulating most of the targets related to NP; HGWD regulates β‐function through TNF, IL‐17, and MAPK signaling pathways. However, this speculation needs further experimental verification.

## 5 CONCLUSION

The analysis showed that 31 compounds and 41 target genes were screened out from five herbs of HGWD. In particular, 15 genes are highly correlated with NP. The major compounds that β‐sitosterol, (+)‐catechin, quercetin, stigmasterol, and kaempferol and the centrical pathways that MAPK, IL‐17, and TNF signaling pathways, which related to central genes such as IL‐6, ICAM1, and CASP3. According to the analysis results, it is speculated that HGWD may play a role in treating NP and provide a reference for subsequent experimental studies to further verify its mechanism of action.

## AUTHOR CONTRIBUTIONS

Bo‐Yan Luo wrote the manuscript, Hong‐Su Zhou and Qiu‐Xia Xiao helped in revising the manuscript, and Yu‐Qi He conceived and reviewed the manuscript. All authors had read and approved the final manuscript.

## CONFLICTS OF INTEREST

The authors declare no conflicts of interest. Qiu‐Xia Xiao is a reviewer of Ibrain but is not involved in the peer review process of this article.

## ETHICS STATEMENT

Not applicable.

## Data Availability

The data sets used and/or analyzed during the current study are available from the corresponding author on reasonable request.
